# Treating non-responders: pitfalls and implications for cancer immunotherapy trial design

**DOI:** 10.1186/s13045-020-0847-x

**Published:** 2020-03-14

**Authors:** Zhenzhen Xu, Yongsoek Park, Ke Liu, Bin Zhu

**Affiliations:** 1 Center for Biologics Evaluation and Research (CBER), U.S. Food and Drug Administration (FDA), 10903 New Hampshire Ave, Silver Spring, MD 20993 USA; 2 Department of Biostatistics, University of Pittsburgh, Pittsburgh, PA 15261 USA; 3 Division of Cancer Epidemiology and Genetics, National Cancer Institute, Bethesda, MD 20892 USA

**Keywords:** Cancer immunotherapy, Dichotomized response, Immuno-oncology trial, Non-proportional hazards pattern, Proportional hazards assumption, Sample size and power calculation

## Abstract

**Background:**

Conventional trial design and analysis strategies fail to address the typical challenge of immune-oncology (IO) studies: only a limited percentage of treated patients respond to the experimental treatment. Treating non-responders, we hypothesize, would in part drive non-proportional hazards (NPH) patterns in Kaplan-Meier curves that violates the proportional hazards (PH) assumption required by conventional strategies. Ignoring such violation incurred from treating non-responders in the design and analysis strategy may result in underpowered or even falsely negative studies. Hence, designing innovative IO trials to address such pitfall becomes essential.

**Methods:**

We empirically tested the hypothesis that treating non-responders in studies of inadequate size is sufficient to cause NPH patterns and thereby proposed a novel strategy, *p-embedded*, to incorporate the dichotomized response incurred from treating non-responders, as measured by the baseline proportion of responders among treated patients *p*%, into the design and analysis procedures, aiming to ensure an adequate study power when the PH assumption is violated.

**Results:**

Empirical studies confirmed the hypothetical cause contributes to the manifestation of NPH patterns. Further evaluations revealed a significant quantitative impact of *p*% on study efficiency. The p-embedded strategy incorporating the properly pre-specified *p*% ensures an adequate study power whereas the conventional design ignoring it leads to a severe power loss.

**Conclusion:**

The p-embedded strategy allows us to quantify the impact of treating non-responders on study efficiency. Implicit in such strategy is the solution to mitigate the occurrence of NPH patterns and enhance the study efficiency for IO trials via enrolling more prospective responders.

## Introduction

**Fig. 1:**
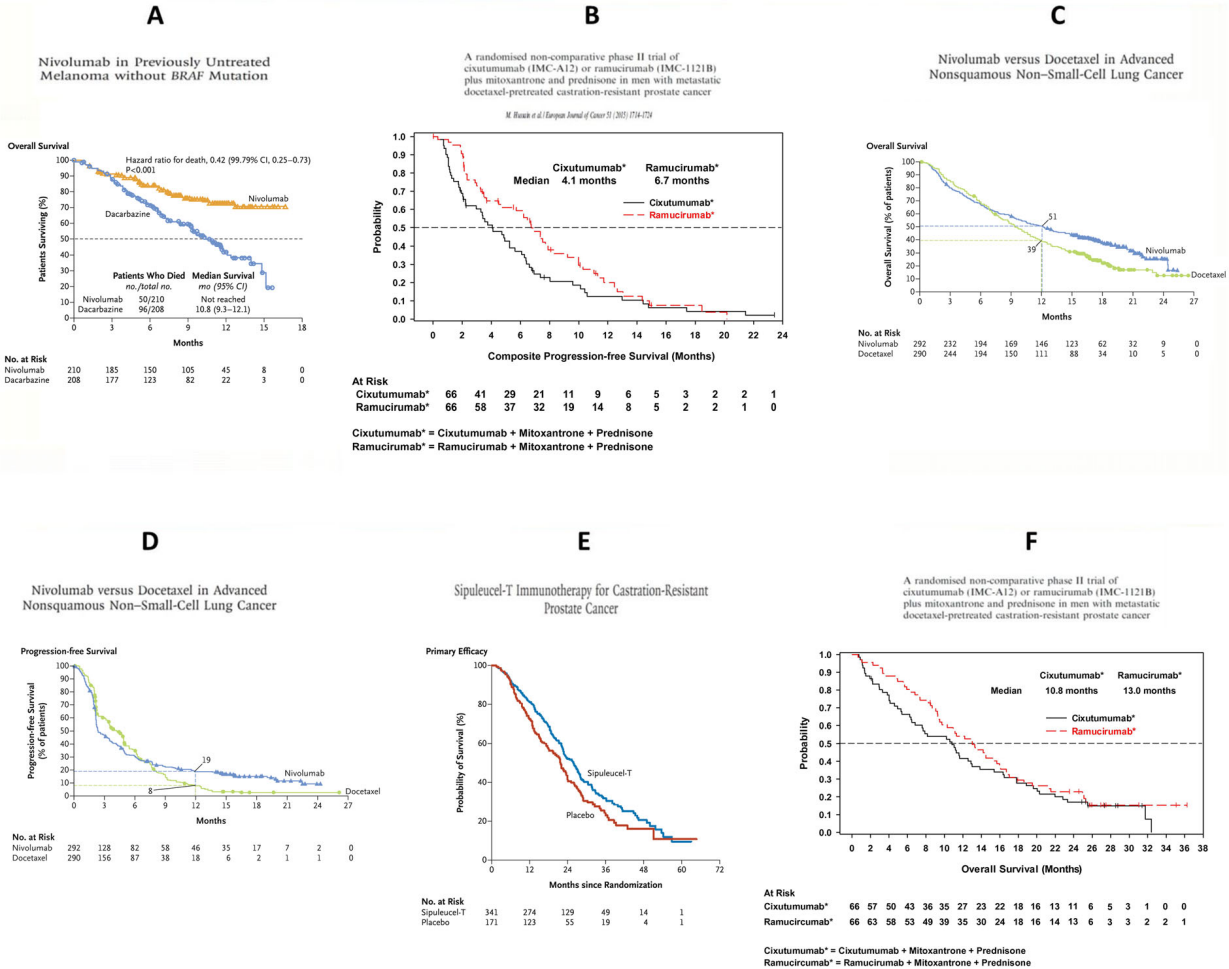
Real study examples on non-proportional hazards (NPH) patterns. **a** Delayed treatment effect pattern: nivolumab in previously untreated melanoma without BRAF mutation (*N* = 418); adapted from C Robert, GV Long, B Brady, C Dutriaux, M Maio, L Mortier, JC Hassel, P Rutkowski, C McNeil, E Kalinka-Warzocha, et al. [1]. **b** Belly-shape diminishing effect pattern: A randomised non-comparative phase II trial of cixutumumab (IMC-A12) or ramucirumab (IMC-1121B) plus mitoxantrone and prednisone in men with metastatic docetaxel-pretreated castration-resistant prostate cancer; adapted from M Hussain, D Rathkopf, G Liu, A Armstrong, WK Kelly, A Ferrari, J Hainsworth, A Joshi, RR Hozak, L Yang, et al. [4]. **c** Crossing hazards pattern: nivolumab versus docetaxel in advanced squamous-cell non-small cell lung cancer (*N* = 582); adapted from J Brahmer, KL Reckamp, P Baas, L Crino, WE Eberhardt, E Poddubskaya, S Antonia, A Pluzanski, EE Vokes, E Holgado, et al. [2]. **d** Delayed effect and crossing hazards combination pattern: nivolumab versus docetaxel in advanced squamous-cell non-small cell lung cancer (*N* = 582); adapted from J Brahmer, KL Reckamp, P Baas, L Crino, WE Eberhardt, E Poddubskaya, S Antonia, A Pluzanski, EE Vokes, E Holgado, et al. [2]. **e** Delayed effect and belly-shape diminishing effect combination pattern: sipuleucel-T immunotherapy for castration-resistant prostate cancer; adapted from PW Kantoff, CS Higano, ND Shore, ER Berger, EJ Small, DF Penson, CH Redfern, AC Ferrari, R Dreicer, RB Sims, et al. [3]. **f** Various belly-shape diminishing effect combination pattern: a randomized non-comparative phase 2 trial of cixutumumab (IMC-A12) or ramucirumab (IMC-1121B) plus mitoxantrone and prednisone in men with metastatic docetaxel-pretreated castration-resistant prostate cancer; adapted from M Hussain, D Rathkopf, G Liu, A Armstrong, WK Kelly, A Ferrari, J Hainsworth, A Joshi, RR Hozak, L Yang, et al. [4]

The unprecedented growth in immuno-oncology (IO) trials has outstripped the development of proper design and analysis strategies. Particularly, a variety of complex survival patterns frequently arise in conventionally designed IO trials with time-to-event endpoints, including the *delayed treatment effect pattern* where Kaplan-Meier (KM) curves for the two treatment groups overlay during the early trial stage (Fig. 1a), the *belly-shape diminishing effect pattern* where the KM curves first separate then join after sufficient follow-up (Fig. 1b), the *crossing hazards pattern* where the KM curve for treatment starts out being worse than that for active control but ends up being better (Fig. 1c), or the *combination patterns* that combine the aforementioned patterns in various fashions (Fig. 1d–f). These complex patterns reveal that the underlying hazard rate of the treatment arm is no longer proportional to that of the control arm over time, violating the proportional hazards (PH) assumption required by the conventional design and analysis strategies. This non-proportional difference in hazards makes most conventionally designed IO studies underpowered or even falsely negative. Hence, how to design tailored and innovative IO studies to mitigate the power loss becomes the question of interest.

There is much recent literature introducing novel trial designs for IO studies with non-proportional hazards (NPH) patterns [5–11]. Most proposals, however, only address a particular category of complex patterns and may not be applicable to other categories. Moreover, none of these proposals provide a solution to avert the occurrence of NPH patterns at the design stage. Hence, to address the question of interest, we should go beyond asking: what is the underlying cause behind various patterns? Many biological and clinical evidences show that the indirect mechanism of action of immunotherapy results in delayed treatment responses, causing the non-proportional hazards. However, would the lag duration manifested in the Kaplan-Meier curves reflect the true delayed duration? Moreover, what are the causes behind the NPH patterns other than the delayed treatment effect pattern, such as the crossing hazards pattern? The prevalence of various patterns seems to indicate that there exist some other causes besides a working mechanism.

In this paper, we first investigated the possible causes behind the NPH patterns and resultant power loss. Inspired by the recognition that cancers are highly heterogeneous diseases [12], we conjecture that the manifestation of such patterns stems in part from treating patients who are not likely to respond to the experimental therapy in studies of limited size. Traditionally designed IO trials often impose very liberal entry criterion and enroll all comers of the target cancer type, likely resulting in a heterogeneous trial population for which only a small subset of patients harbor immunotherapy-sensitive tumors [13] and would respond to the immunotherapy once being treated [14–18] (responders), whereas the rest of patients would not respond (non-responders). In this article, the responders are broadly defined as those subjects who can benefit from the IO treatment and achieve improved overall survival (OS) due to reduced hazards. Thus, the hazard ratio (HR) between responders and controls would be reduced from one attributable to treatment whereas the ratio between non-responders and controls would remain at one. When it comes to calculating the sample size, trialists normally over-specify the treatment effect size by targeting only the responders and ignoring the existence of non-responders in the treatment arm, inadvertently unaware that the beneficial effect of treatment on the former has been negated by the null effect on the latter. Consequently, such a study would end up underpowered and possibly fail to detect a truly effective immunotherapy, if any. Likewise, when the comparison between treatment and control is depicted using a KM plot, a variety of NPH patterns would likely be manifested, since the HR measuring the average effect of responders and non-responders in the treatment group versus the controls is driven by the proportion of responders and this proportion is constantly modified over the course of study.

Although NPH patterns result partially from treating non-responders, eliminating it remains difficult due to the challenge in identifying the prospective responders definitely. Predictive biomarkers play a critical role in selecting therapeutic-sensitive responding patients for IO treatment; the existing biomarkers, however, cannot predict a particular patient’s therapeutic response with certainty [19, 20], and in many malignancies, the biomarkers are yet to be available for responder selection. Despite the individual responder membership is unknown a priori, however, the chance of an individual patient responding to a given treatment, can be estimated by the proportion of responders in the treatment group at the baseline, *p*%, via prior studies. An innovative analysis procedure can acknowledge and incorporate the response dichotomy, incurred from treating non-responders and measured by *p*%, to ensure an adequate study power.

This idea leads to the proposed p-embedded strategy, including the *p-embedded sample size* and *power calculation algorithm* for design, *p-embedded re-randomization test* for hypothesis testing and *p-embedded EM algorithm* for treatment effect estimation. The p-embedded strategy involves essentially a mixture model where the treatment group is a mixture of responders and non-responders although the individual responder membership is unknown. Such mixture model is different from the existing mixture models including cure model [21] for which all treated patients respond with a subset of them cured. The proposed method has three advantages. First, it ensures an adequate study power to detect a potentially effective therapy when a dichotomized treatment response is present. Second, it explicitly reveals that treating more responders would dramatically reduce the study size and/or shorten the trial duration. Third, it provides a remedy to mitigate the occurrence of NPH patterns. Although the response dichotomy of IO studies has been well recognized and the highly responsive immunogenic subtypes for various cancer types have been identified [22–24], how to take advantage of such advances in the trial design and subsequent analysis has yet to be thoroughly explored, which is therefore the focus of this article.

## Methods

### The effect of response dichotomy and inadequate sample size on the emergence of NPH patterns

We first conducted a simulation study to demonstrate that low proportion of responders *p*% combined with inadequate sample size *N* could be sufficient to cause NPH patterns. Assuming the responders accounting for 20% of treated patients at baseline, we simulated 100 randomized trials of 200 patients each. Within each trial, patients were allocated to the treatment and control arms at 1:1 ratio. The objective was to compare the difference in overall survival (OS) between the two arms, where the median survival time for responders was anticipated to be 3.3 times longer than that of non-responders or controls. The details of the simulation setting are provided in Additional file 1. We depicted the simulated data with KM curves, inspected the resultant patterns visually and summarized them in terms of proportions of trials falling into each aforementioned pattern category. To contrast the joint effect of *p*% and *N* on NPH patterns, the same analysis was repeated when *p*% was increased from 20 to 90% and/or *N* from 200 to 2000.

### Incorporating response dichotomy into the design and analysis of IO trials

To properly incorporate response dichotomy into the design and analysis of IO studies, we first developed the p-embedded re-randomization test for hypothesis testing and *p-embedded expectation maximization* (EM) *algorithm* for treatment effect estimation. Particularly, we assumed that at baseline *p*% of treated patients would respond to the experimental therapy and their survival times follow a piecewise constant exponential distribution, for which the hazard rates vary from *h*_*C*_ to *h*_*T*_ at the lag time *t*^∗^ since randomization. The survival times of non-responders and controls would follow a regular exponential distribution with a constant rate *h*_*C*_. We considered the administrative censoring mechanism under which patients would either die during the course of the study or be censored at the end of the study.

Since the latent response status of each individual treated patient was unknown, we resorted to the expectation-maximization (EM) algorithm [25, 26] for estimating the treatment effect measured by HR ${\lambda}_p=\frac{h_T}{h_C}$, while incorporating the aggregated information on proportion of responders into the estimation procedure. The p-embedded EM algorithm worked iteratively by the E-step and M-step, with E-step guessing the latent responder status of each treated patient under the constraint that the overall proportion of responders among treated was *p*% and M-step using those guesses to estimate the parameters *h*_*C*_ and *λ*_*p*_ (or *h*_*T*_) by maximizing the likelihood of pre-specified survival distributions. The parameter estimates from M-step further refined the guesses of latent responses in the next E-step and the refined guesses in turn fine-tuned the parameter estimates in the next M-step. The iteration continued until the algorithm converged, and the treatment effect estimate at the convergence, ${\hat{\lambda}}_p$, was selected as the outcome of p-embedded EM algorithm.

To test the significance of estimated treatment effect and obtain a valid inference, a non-parametric re-randomization test embedded with *p*% was adopted, due to the fact that the overall sample size was limited and only a small subset of treated patients could contribute to the detection of treatment effect; thus, the asymptotic distribution of ${\hat{\lambda}}_p$ obtained from the EM algorithm may not well approximate the true null distribution. Specifically, for any pre-specified *p*%, the p-embedded re-randomization test worked by first calculating the observed test statistic, ${\hat{\lambda}}_p^{obs}$, using p-embedded EM algorithm on the observed data, then fixing the data at their observed values, regenerating treatment assignments for the entire trial, and computing the test statistic ${\hat{\lambda}}_p^{shuf}$ corresponding to those re-shuffled assignments using the same p-embedded EM algorithm. This process was repeated for a large number of times, and a *p* value was computed as the proportion of re-randomized trials whose test statistic ${\hat{\lambda}}_{p\  shuf}$ was at least as extreme as that of the observed assignments ${\hat{\lambda}}_p^{obs}$.

Based upon the proposed testing procedure, we developed the corresponding p-embedded sample size and power calculation algorithm through numeric simulations. Given the target power, treatment effect size *λ*_*p*_, baseline hazard *h*_*C*_, and baseline proportion of responders among treated *p*%, the proposed algorithm involved a grid search of sample size over a plausible range. Each candidate sample size was evaluated for its empirical power by repeating the following steps a large number of times:

Step 1. Randomized patients to the treatment and control arms at 1:1 ratio to obtain the observed treatment assignments;

Step 2. Generated the observed survival outcome for each patient assuming *h*_*C*_ and *λ*_*p*_ and determined the censoring status under the administrative censoring mechanism;

Step 3. Carried out the p-embedded re-randomization test as described in the previous paragraph to compute the associated *p* value;

Step 4. Step 1 to 3 were repeated for a large number of times and for each time, the *p* value was recorded.

The estimated empirical power for a given candidate sample size was the proportion of *p* values that were less than or equal to 0.05. Among the candidate sample sizes, the one whose empirical power was closest to the target was then selected as the sample size required to achieve the target power, *N*.

### Robustness against the mis-specification risk of *p*%

Apparently, the p-embedded design and analysis procedure depends critically upon the pre-specification of *p*%; but in practice, the true value of *p*% is unknown. It is thus critical to address the robustness property of the p-embedded strategy against the mis-specification risk under various scenarios. To this end, we first computed the required sample size *N* using the p-embedded design when *p*% was over-specified or under-specified from the truth as *p*^*m*^%. Next, given *N*, we calculated the empirical power that the p-embedded analysis procedure could actually achieve based on the mis-specified *p*^*m*^%. For comparison purpose, we also evaluated the conventional design ignoring response dichotomy. To calculate the empirical power under the mis-specification scenarios, the survival outcomes for the responders and non-responders/controls were simulated under the true *p*%, but the *p* values were computed under the mis-specified *p*^*m*^% using the p-embedded re-randomization test. The aforementioned process was repeated 1000 times to obtain the empirical power.

### Software

To facilitate the implementation of the p-embedded strategy in practice, we have developed a software “Immunotherapy.Design” that allows users to perform the p-embedded sample size and power calculation, hypothesis testing as well as treatment effect estimation. The software is freely available at GitHub https://github.com/wheelerb/Immunotherapy.Design.

### Trial example

To illustrate the p-embedded strategy for the design and analysis of IO trials, consider a phase 3 study with the target power at 80% and type I error rate at 0.05. Suppose the enrollment rate of 0.53 subject per day, delayed duration of one month and overall study duration of 5 years, the hazard ratio for the treated responders versus controls is pre-specified to be 0.6, and the responders account for 60% of treatment group (*p*% = 60%). Targeting 80% power, the p-embedded sample size and power calculation algorithm built in the Immunotherapy.Design software conducts a grid search of sample size over a plausible range between 100 and 300 and determines 275 subjects are required to achieve the target power. Further suppose that a hypothetical trial is conducted with 275 patients enrolled, whose time-to-event data were simulated from a piecewise constant exponential distribution for the treated responders and from a regular exponential distribution for the treated non-responders/controls, respectively. Applying the p-embedded EM algorithm on the hypothetical trial gives an estimate of hazard ratio between responders and controls at 0.595, very close to the underlying truth of 0.60 used to generate the hypothetical trial data, and performing the p-embedded re-randomization test evaluating the significance of treatment effect leads to a *p* value of 0.023.

## Results

### Low proportion of responders plus inadequate sample size could cause NPH patterns

The exploratory study, which simulated 100 randomized trials with different *p*% and *N*, revealed that low proportion of responders plus inadequate sample size could cause NPH patterns to arise. Among the 100 clinical trials of 200 patients each with a *p*% of 20%, a disproportionate portion (89%) manifested NPH patterns of various kinds: 27% of delayed treatment effect pattern with a prolonged (or shortened) lag of longer than (or shorter than) the underlying truth of 3 months, 12% of belly-shape diminishing effect pattern, 9% of crossing hazards pattern, and 41% of combination pattern. In contrast, if *p*% increased to 90%, the overwhelming majority of simulated trials displayed a clear separation of survival curves between arms right after the 3-month lag and only 20% of trials demonstrated diverse NPH patterns. It is interesting to note that *N* played a critical role in the causal impact of *p*% on NPH patterns. As *N* increased, the impact of *p*% varnished. Among trials simulated under a small *p*% of 20%, a sample size of 2000 patients resulted in 23% of trials involving NPH patterns, whereas *N* of 200 led to 89% of them showing complex patterns. This empirical analysis shedded light on the driving forces behind NPH patterns and supported our conjecture. It thus also provided a remedy to avert the occurrence of NPH patterns by either treating more responders or increasing *N* or both.

### Impact of response dichotomy on study efficiency

The p-embedded sample size and power calculation algorithm quantifies the impact of response dichotomy on study efficiency through the relationship between *p*% and *N* under a given target power (Table 1). For comparison, *N* was computed by the p-embedded and conventional designs, respectively. To detect a 70% increase in median survival time for treated subjects relative to the controls with a desired power, the conventional design required 27 patients, whereas the p-embedded design entailed 269 patients when only 20% of treated patients respond. The simulation study confirmed that 269 patients achieved 80% of power empirically based on the p-embedded re-randomization test whereas 27 patients only provided 8.81% of empirical power based on the conventional log-rank test. This evaluation reveals that ignoring the dichotomized treatment response in the design and analysis strategy would result in a severe underestimate of sample size and consequently a serious loss of power, whereas recognizing such dichotomy yields a proper increase in sample size so that an adequate and well-controlled study can be ensured. Although one may argue that a randomized trial with 27 patients is apparently uncommon in practice, we aim to use such a study to illustrate the detrimental effect of ignoring *p*% in IO trials with a large treatment effect size for responders (HR = 0.3) to be detected.

**Table 1:** Impact of treating non-responders and the incurred response dichotomy on the required sample size (*N*) and empirical power (EP). Response dicotomy is measured by the proportion of responders (*p*%) among the treatment arm at baseline. Target power is 80%. Lag duration is 1 month. Hazard ratio for responding patients is 0.3. Total study duration is 5 years. Enrollment rate is 0.53 subjects/day

	P-embedded design		Conventional design	
*p* (%)	N	Empirical power	N	Empirical power
20	269	80%	27	8.81%
30	137			13.39%
40	89			18.58%
50	68			25.73%
60	52			32.81%

If *p*% increases from 20 to 60%, *N* required by p-embedded design plummeted from 269 to 52 patients. Such a precipitous decrease implies that if only a minority of treatment arm respond (e.g., 20%), even to detect a highly effective treatment regimen with the desired power requires a very large sample size, as opposed to that required when a majority of treatment arm responds (e.g., 60%). This finding has an intuitive implication. To achieve the desired power under a dichotomized treated population with a small subset of latent responders, investigators would need to enrich the study without knowing what to enrich, leaving the magnitude of enrichment inefficiently large. In contrast, treating more, if not all, responders, can significantly reduce the study size and enhance the study efficiency, which underscores the value of precision design for immunotherapy studies.

Next, we explored how trial parameters, such as the magnitude of treatment effect, trial duration as well as lag duration, affect the relationship between response dichotomy and study efficiency.

### Magnitude of treatment effect

**Fig. 2:**
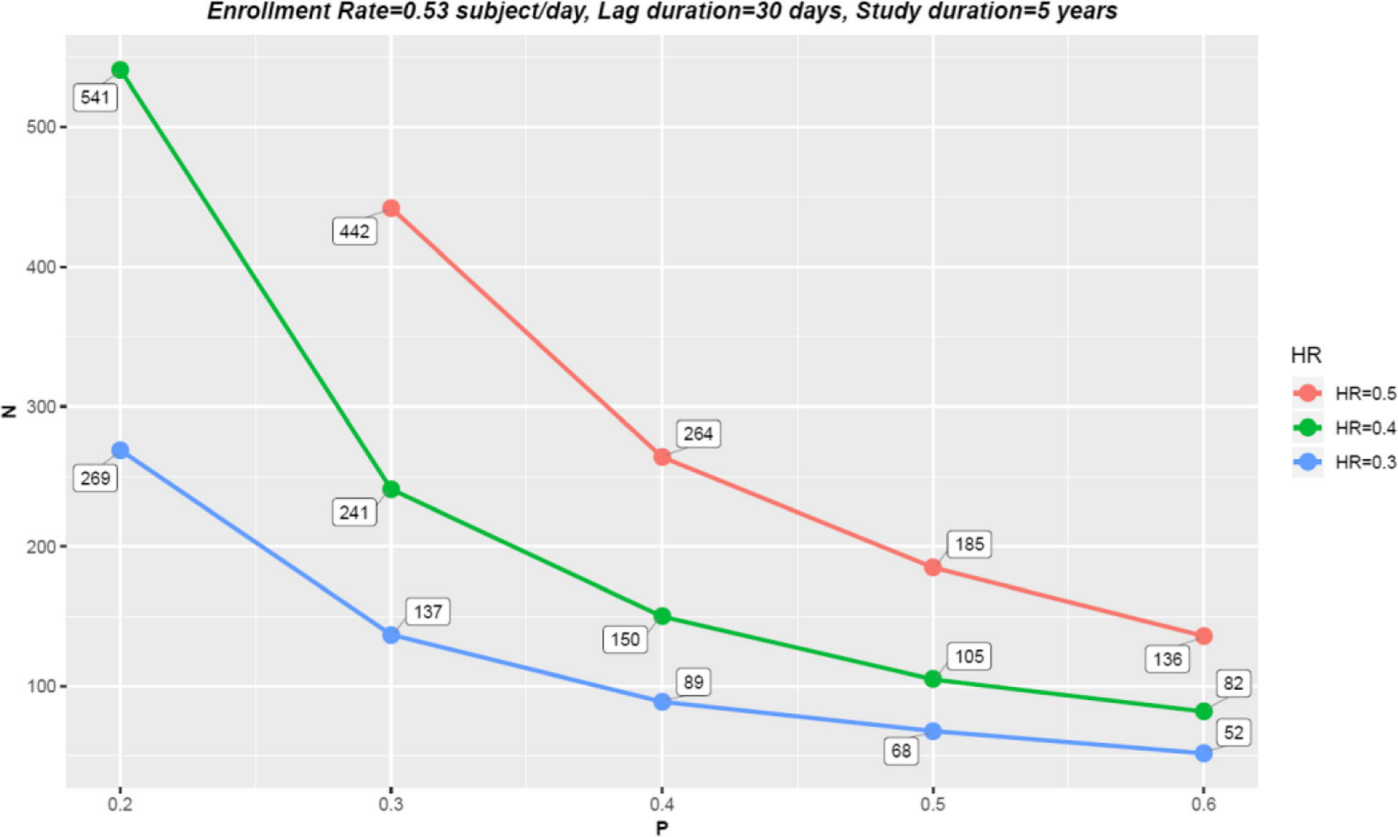
The impact of response dichotomy on study efficiency as magnitude of treatment effect (measured by hazard ratio) varies

As the magnitude of treatment effect varies, similar trends could be observed between the proportion of responders and the required sample size (Fig. 2). The association that the lower the proportion of responders, the larger the required sample size became more apparent when the treatment effect got smaller. For instance, when the post-lag hazard ratio for responders was 0.3, a decrease in *p*% from 60 to 20% resulted in an increase in *N* from 52 to 269; when the hazard ratio increased to 0.4, the same variation in *p*% led to a dramatic inflation in *N* from 82 to 541. As the hazard ratio became 0.5, an even more extreme trend was present where, if *p*% was 20%, no matter how large *N* rose to, the study could no longer achieve the desired power given the same enrollment rate and study duration. The reason was because the subjects who were enrolled late to the study could no longer be sufficiently followed up until their event times before the study ends. Thus, the strategy to salvage the loss of power due to low proportion of responders via increasing the sample size only worked when the treatment effect size was relatively large and the study duration was reasonably long. On the contrary, when the effect size was moderate or the study duration could not be further extended, enhancing the proportion of responders *p*%, that is, enrolling more responders, seemed to be the only sensible solution to achieve the desired power.

### Trial duration

Trial duration plays a critical role in rescuing the power loss when the response dichotomy is severe. As Table 2 illustrates, a study with 20% treated responders was impossible to achieve the desirable power given 3 years of duration and 0.53 subjects/day enrollment rate. In order to salvage the power loss, the study duration needed to be prolonged to allow sufficient follow-up time for patients who were enrolled late. Table 2 also reveals an interesting interplay among the trial duration, required sample size *N* and proportion of responders *p*%. In general, as the trial duration extended, achieving the target power required less sample size, given a fixed value of *p*%. As *p*% increased, such impact of trial duration on sample size diminished and the study remained efficient even under a short duration. For instance, when *p*% became as large as 60%, *N* converged to approximately 52 patients regardless of the study duration. This finding once again highlights the benefits of enhancing *p*% in conducting faster and smaller trials.

**Table 2:** Impact of response dichotomy on the sample size required to achieve the target power by study duration. Target power is 80%. Lag duration is 1 month. Hazard ratio for responding patients is 0.3. Enrollment rate is 0.53 subjects/day

*p* (%)	Sample size required to achieve the target power		
	Study duration		
	3 years	4 years	5 years
20	NA	313	269
30	186	153	137
40	108	96	89
50	76	70	68
60	55	52	52

### Lag duration

**Fig. 3:**
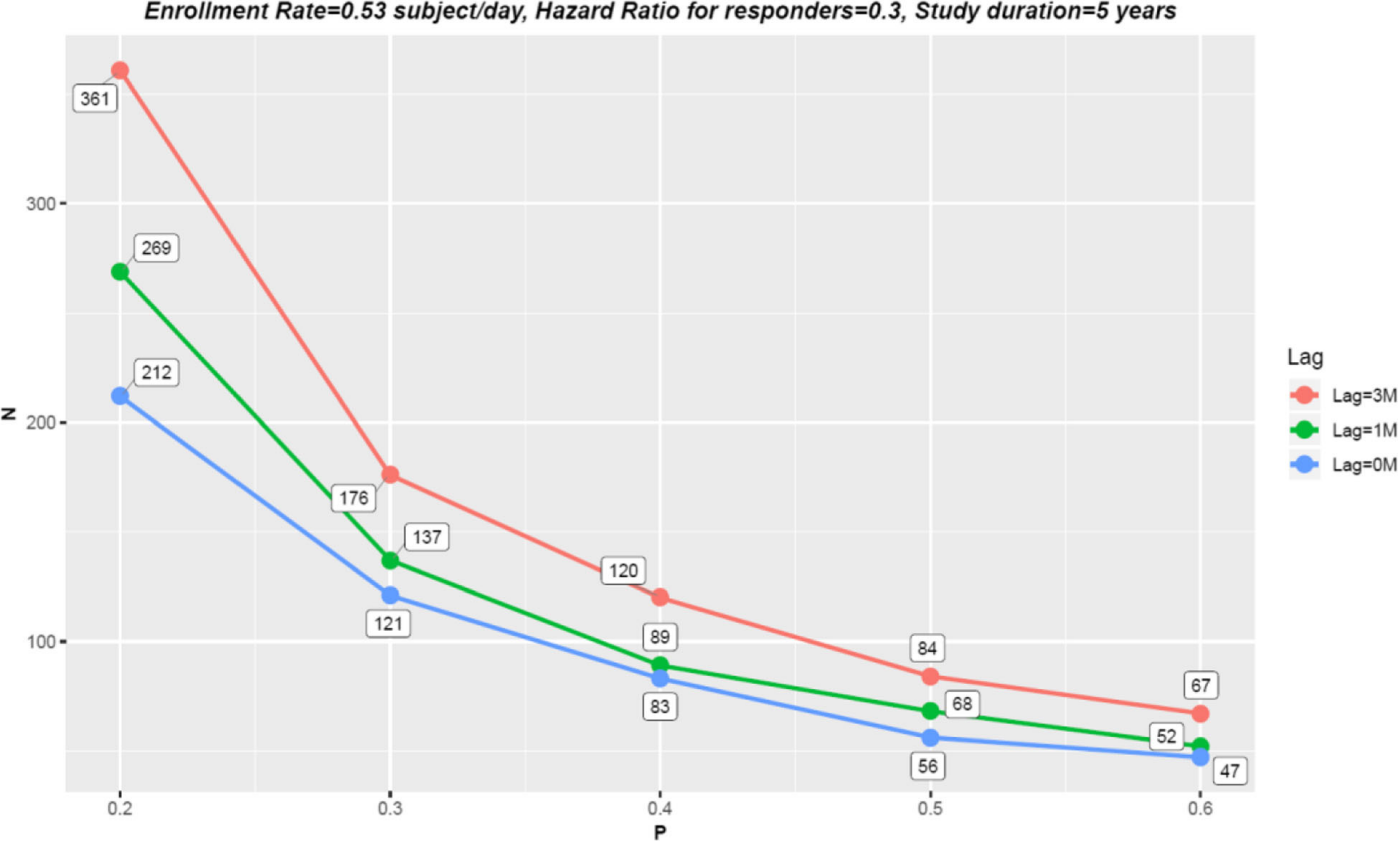
The impact of response dichotomy on study efficiency as duration of treatment lag varies

When the treatment time-lag effect is present, an IO study generally requires more sample size to achieve the target power. Figure 3 demonstrates that given the same *p*%, the longer the lag, the larger the sample size. As *p*% varied, the trajectory of *N* remained roughly similar regardless of the lag duration, although the inflation in *N* due to a decrease in *p*% seemed slightly more apparent under a longer lag.

### Impact of mis-specifying *p*%

Table 3 illustrates that over-specifying *p*% from the truth would result in underpowered studies but under-specifying *p*%, overpowered ones, and the deviation from the target power due to the mis-specification varnished as *p*% increased. For example, the p-embedded design based on a mis-specified *p*% at 60% claimed that 52 patients were sufficient to achieve the target power at 80%. If the true *p*% fell below the specified (*p*% = 50%) or above it (*p*% = 70%), the empirical study revealed that the p-embedded analysis procedure given 52 patients could actually achieve 71.4% or 88.0% of power, respectively, where the deviation from the target power was less than 10%. In contrast, when the true *p*% was small, even the same magnitude of mis-specification in *p*% could result in a more severe deviation in power from the target. For instance, if *p*% was mis-specified to 20% but the truth lied at 10% or 30%, the resulting study designed and analyzed by p-embedded strategy gave rise to 44.9% or 95.2% of power, where the deviation from the target power was more than 10%. By contrast, the conventional study led to an even more severe loss of power (empirical powers were 6.61% or 13.39%).

**Table 3:** The impact of mis-specifying *p*% on the design and analysis. At the design stage, the sample size required to achieve the target power is calculated using the conventional design ignoring response dichotomy and the p-embedded design recognizing response dichotomy but mis-specifying the true *p*^∗^% to be *p*^*m*^%. Given the sample size calculated, the empirical power (EP) is evaluated under the true response dichotomy *p*^∗^%. Target power is 80%. Lag duration is 1 month. Hazard ratio for responding patients is 0.3. Total study duration is 5 years

P-embedded design						Conventional design				
Design		Analysis				Design	Analysis			
Mis-specifying p^*^		Over-specifying p^*^ = *p*^m^ − 10		Under-specifying p^*^ = *p*^m^ + 10		Ignoring p^*^				
*p*^m^ (%)	*N*	p^*^ (%)	EP	p^*^ (%)	EP	*N*	p^*^ (%)	EP	p^*^ (%)	EP
20	269	10	44.90%	30	95.20%	27	10	6.61%	30	13.39%
30	137	20	59.30%	40	88.90%		20	8.81%	40	18.58%
40	89	30	63.70%	50	89.20%		30	13.39%	50	25.73%
50	68	40	70.30%	60	89.00%		40	18.58%	60	32.81%
60	52	50	71.40%	70	88.00%		50	25.73%	70	40.93%

Three conclusions can be drawn. First, the study power based on the p-embedded strategy can be severely overestimated when *p*% is very small and lies below the specified. Second, despite being underpowered under certain circumstances, the proposed approach accounting for the dichotomized response is still much more powerful than the conventional method ignoring it completely. Intuitively thinking, ignoring the response dichotomy could be regarded as over-specifying *p*% to 100% by assuming all enrolled patients could uniformly respond to a given therapy, a severe over-specification that would result in a serious power loss. Third, as *p*% increases, the mis-specification risk is alleviated, implying that increasing *p*% by treating more responders is helpful to guard against the mis-specifying risk. In practical application of the p-embedded strategies, it is essential to conduct sensitivity analysis by evaluating various plausible values of *p*%.

## Discussions

Treating non-responders and ignoring the response dichotomy incurred are a potential pitfall in designing IO trials. Specifically, response dichotomy combined with inadequate study size would likely give rise to NPH patterns in Kaplan-Meier curves, undermine the study power and make the treatment effect difficult to detect. Although eliminating the response dichotomy by identifying individual responding patient remains challenging, properly pre-specifying the proportion of responders *p*% among a group of patients with certain characteristics can be feasible, and that information can be taken advantage of to ensure an adequate power. Hence, we have developed the p-embedded strategy for the design and analysis of IO trials, which allows us to quantify the impact of treating non-responders on study characteristics and properly inflate the sample size to salvage the potential power loss. Our findings reveal that if the treatment group consists of only a small subset of latent responders, salvaging the power loss requires enriching the study without knowing what to enrich, thus leaving the magnitude of enrichment inefficiently large or even infeasible. Conversely, if more prospective responders would be enrolled so that *p*% is increased, the NPH patterns can be mitigated and study efficiency dramatically improved as we demonstrate quantitively in this article. These results underscore the importance and benefits of precision design for immunotherapy trials. It is worth noting that the benefits from properly enriching the subset of responders would be associated with not only IO agents but also non-IO agents.

In this article, the responders are broadly defined as those subjects who can benefit from the IO treatment by achieving improved OS due to reduced hazards, so *p*% is generally larger than the objective response rate. From the clinical perspective, the responders could include patients who can achieve complete response (CR), partial response (PR) as well as durable stable disease (SD) which is also clinically meaningful and can translate to improved OS, per RECIST criteria. Such definition of responders has pros and cons, respectively. The pros are that it may fully capture the OS benefits and is not restricted to objective response, as not all survival benefits for IO therapy can be adequately captured by objective response. The disadvantage is that it creates added challenge for the investigators to pre-specify the proportion of prospective subjects who can take advantage of improved OS benefits, *p*%.

How to properly pre-specify *p*% and how to enroll a patient population with large *p*% are the critical questions to be addressed when applying the proposed strategy. In practice, pre-specifying *p*% and HR for responders is challenging yet feasible based on early-phase studies. As described in the US Food and Drug Administration Guidance on Clinical Considerations for Therapeutic Cancer Vaccines [27], a randomized phase 2 trial is highly recommended prior to the transition to confirmative trials to help determine the appropriate sample size and treatment effect size. Given the study results of a randomized phase 2 trial with the target cancer type, one can modify the p-embedded EM algorithm to estimate not only the HR of responders but also *p*%. If the confirmatory study enrolls patients with same characteristics, the estimates from phase 2 can help to properly pre-specify *p*% for phase 3. More importantly, when a predictive biomarker is available, it can facilitate the selection of study population with a larger *p*%. For instance, if patients’ PD-L1 expression [28] levels are collected in phase 2, then the p-embedded EM algorithm can be applied to estimate *p*% and HR for PD-L1 expression positive or negative subgroups, respectively. Investigators can then enroll patients who are PD-L1 expression positive exclusively for phase 3, so that the confirmatory trial could have a patient population with a higher likelihood of therapeutic response (i.e., larger *p*%) as compared with the one enrolling patients of all subtypes. At present, it is not feasible to predict definitively a particular patient’s therapeutic response based on the existing biomarkers [19, 20], which however makes the p-embedded strategy particularly useful as it incorporates the uncertainty in patients’ therapeutic responses into the design and analysis to salvage power loss. With the refinement of existing biomarkers [29, 30] and identification of novel biomarkers [31], more rational use of biomarkers could be ensured to direct the selection of trial population who is more likely to benefit, making it feasible to enhance the study efficiency of IO trials. Ultimately, it helps to administer the immunotherapy to the prospectively responding patients in the real world. Artificial Intelligence (AI) may help improve the efficiency of IO trials conducted using the p-embedded strategy via enrolling a study population with a larger *p*%. When large-scale prior studies become available, AI may help identify the biomarkers [32] to predict the responses of individual patients to IO treatments [30, 33, 34] so that therapeutic-sensitive patients can be enrolled and a study population with a larger *p*% can be obtained.

Despite the many benefits of p-embedded strategy can provide, the proposed approach is only suitable within certain scope. For instance, p-embedded is not suitable for a single-arm study or a study for which patients can homogeneously benefit from the therapeutic agent (such as *p*% is close to 100%). In the extreme case when *p*% is impossible to elicit due to the lack of any prior information, the p-embedded strategy cannot be adopted or perform well. More likely, insufficient knowledge about *p*% is availabe and there are chances of mis-specifying *p*%. In this case, to control the risk of mis-specification within a reasonable range, historical studies, pilot data, and good biological and medical judgment on the mechanism of action of therapeutic agent are recommended to utilize, appropriately, for study design. In addition, sensitivity analysis should always be conducted to explore various plausible values of *p*%. Another limitation of the p-embedded strategy is the robustness of the parametric assumption as the baseline hazard is modeled parametrically via an exponential distribution. While acknowledging the limitation, we consider that the parametric model has its merit, as it is sufficient to illustrate the key idea that quantifying the impact of treating non-responders is essential for IO trial design and reducing incurred response dichotomy can enhance the study efficiency significantly. A more robust non-parametric approach is currently under development for future research.

## Conclusions

In summary, we identified that NPH patterns in IO trials are largely caused by treating non-responders and proposed a first-of-its-kind strategy, p-embedded, to address the incurred dichotomized treatment response. The p-embedded strategy makes two significant contributions: first, it ensures an adequate study power of IO trials by properly pre-specifying response dichotomy, which significantly reduces falsely negative IO studies; second, it provides a solution to enhance the IO trial efficiency and mitigates the occurrence of NPH patterns by enrolling more prospective responders identified via prior studies, which outlines a path towards precision immunotherapy.

## Supplementary information

**Additional file 1:** A simulation study to investigate the cause of NPH patterns.

## Data Availability

The paper does not involve any real dataset.

## Abbreviations

EM: Expectation-maximization
IO: Immuno-oncology
KM: Kaplan-Meier
NPH: Non-proportional hazards
PH: Proportional hazards
