# Supplementary material for: Treating non-responders: pitfalls and implications for cancer immunotherapy trial design
Source: J Hematol Oncol. 2020 Mar 14;13:20. doi: 10.1186/s13045-020-0847-x (PMC7071722; doi:10.1186/s13045-020-0847-x)
Supplement: Supplementary file 1 — Additional file 1: A simulation study to investigate the cause of NPH patterns. [file 13045_2020_847_MOESM1_ESM.docx]

**Supplementary Material**

**Treating non-responders: pitfalls and implications for cancer immunotherapy trial design**

Zhenzhen Xu, Yongsoek Park, Ke Liu, Bin Zhu

**Supplementary Methods**

*The effect of response dichotomy and inadequate sample size on the emergence of NPH patterns*

We first conducted a simulation study to demonstrate that low proportion of responders $p\%$ combined with inadequate sample size $N$ could be sufficient to cause NPH patterns. Assuming the responders accounting for 20% of treated patients at baseline, we simulated 100 randomized trials of 200 patients each. Within each trial, patients were allocated to the treatment and control arms at 1:1 ratio. The objective was to compare the difference in overall survival (OS) between the two arms. With the enrollment rate of 0.53 subjects per day and overall study duration of three years, we built in a 3-month treatment time-lag effect, as the indirect mechanism-of-action of immune agent likely caused a time lag to manifest its treatment effect[1, 2]. We assumed that the duration of lag and magnitude of post-lag treatment effect were homogeneous across responders. Suppose that 90% of patients could survival beyond the time lag and among them, the median survival time for responders was anticipated to be 3.3 times longer than that of the non-responders or controls. Next, we depicted the simulated data with KM curves, inspected the resultant survival patterns visually and summarized these patterns in terms of proportions falling into each aforementioned pattern category. To contrast the effect of joint effect of $p\%$ and $N$ on NPH patterns, the same analysis was repeated when $p\%$ is increased from 20% to 90% and/or $N$ from 200 to 2000.

References

1. Anagnostou VaY, Mark and Hansen, Aaron R and Wang, Hao and Verde, Franco and Sharon, Elad and Collyar, Deborah and Chow, Laura QM and Forde, Patrick M. Immuno-oncology trial endpoints: capturing clinically meaningful activity. Clinical Cancer Research 2017:4966-4969.

2. Finke LHaW, Kerry and Blumenstein, Brent and Rudolph, Natalie S and Levitsky, Hyam and Hoos, Axel. Lessons from randomized phase III studies with active cancer immunotherapies--outcomes from the 2006 meeting of the Cancer Vaccine Consortium (CVC). Vaccine 2007:B97--B109.
